# Strain-encoded cardiac magnetic resonance imaging: a new approach for fast estimation of left ventricular function

**DOI:** 10.1186/s12872-019-1031-5

**Published:** 2019-03-05

**Authors:** Tomas Lapinskas, Victoria Zieschang, Jennifer Erley, Lukas Stoiber, Bernhard Schnackenburg, Christian Stehning, Rolf Gebker, Amit R. Patel, Keigo Kawaji, Henning Steen, Remigijus Zaliunas, Sören J. Backhaus, Andreas Schuster, Marcus Makowski, Sorin Giusca, Grigorious Korosoglou, Burkert Pieske, Sebastian Kelle

**Affiliations:** 1 Department of Internal Medicine / Cardiology, German Heart Center Berlin, Augustenburger Platz 1, 13353 Berlin, Germany; 2 Department of Cardiology, Medical Academy, Lithuanian University of Health Sciences, Kaunas, Lithuania; 3 Philips Healthcare, Hamburg, Germany; 4 Department of Medicine, University of Chicago, Chicago, IL USA; 5 Department of Internal Medicine / Cardiology, Marienkrankenhaus Hamburg, Hamburg, Germany; 6 Department of Cardiology and Pneumology, University Medical Center, Georg-August University, Göttingen, Germany; 7 Department of Cardiology and Vascular Medicine, GRN Hospital Weinheim, Weinheim, Germany; 8 Department of Internal Medicine / Cardiology, Charité Campus Virchow Clinic, Berlin, Germany; 9 DZHK (German Centre for Cardiovascular Research), Partner Site Berlin, Berlin, Germany; 10 DZHK (German Centre for Cardiovascular Research), Partner Site Göttingen, Göttingen, Germany; 11 Department of Cardiology, Royal North Shore Hospital, The Kolling Institute, Northern Clinical School, University of Sydney, Sydney, Australia; 12 Department of Radiology, Charité Campus Virchow Clinic, Berlin, Germany

**Keywords:** Cardiac magnetic resonance, Strain-encoded imaging, Cine imaging, Left ventricular function

## Abstract

**Background:**

Recently introduced fast strain-encoded (SENC) cardiac magnetic resonance (CMR) imaging (fast-SENC) provides real-time acquisition of myocardial performance in a single heartbeat. We aimed to test the ability and accuracy of real-time strain-encoded CMR imaging to estimate left ventricular volumes, ejection fraction and mass.

**Methods:**

Thirty-five subjects (12 healthy volunteers and 23 patients with known or suspected coronary artery disease) were investigated. All study participants were imaged at 1.5 Tesla MRI scanner (Achieva, Philips) using an advanced CMR study protocol which included conventional cine and fast-SENC imaging. A newly developed real-time free-breathing SENC imaging technique based on the acquisition of two images with different frequency modulation was employed.

**Results:**

All parameters were successfully derived from fast-SENC images with total study time of 105 s (a 15 s scan time and a 90 s post-processing time). There was no significant difference between fast-SENC and cine imaging in the estimation of LV volumes and EF, whereas fast-SENC underestimated LV end-diastolic mass by 7%.

**Conclusion:**

The single heartbeat fast-SENC technique can be used as a good alternative to cine imaging for the precise calculation of LV volumes and ejection fraction while the technique significantly underestimates LV end-diastolic mass.

## Background

Currently, cine cardiac magnetic resonance (CMR) is the accepted standard of reference for quantification of ventricular volumes, mass and function [1]. According to recent recommendations quantification of left ventricular (LV) volumes is performed using manual contouring of the endocardial and epicardial surface from multiple short-axis planes and LV ejection fraction (EF) and mass are calculated. Although automated contouring algorithms are available the majority of dedicated analysis software still relies on human interaction at least in clinical practice. In recent years, important improvements in CMR techniques have significantly reduced scan time, necessary to image the entire heart, while the duration of semiautomated volumetric analysis did not change significantly and takes up to five minutes [2].

The aim of this pilot study was to assess the ability and accuracy of recently proposed fast-SENC technique to estimate LV volumes, ejection fraction and end-diastolic mass.

## Methods

### Study population

We prospectively invited 35 subjects (12 healthy volunteers and 23 patients with known or suspected coronary artery disease) to participate in this single center study. The coronary artery disease (CAD) was confirmed by previous quantitative coronary angiography. The study complies with the Declaration of Helsinki and was approved by the Ethics Committee of the Charité–Universitätsmedizin Berlin. All individuals were able to give written informed consent before entering the study.

### Cardiac magnetic resonance

Study protocol and design details have been previously published [3]. All CMR examinations were performed on a 1.5 T MRI scanner (Achieva, Philips Healthcare, Best, The Netherlands) using a 5-channel phased array receiver coil in the supine position. All study participants were scanned using an identical imaging protocol.

The study protocol included an initial survey to define imaging planes. Cine images were derived using balanced steady state free precession (bSSFP) sequence with short periods of breath-holding in three LV long-axis planes. Short-axis cine images were acquired and used to calculate LV volumes, mass and ejection fraction. The following parameters were used: repetition time (TR) = 3.3 ms, echo time (TE) = 1.6 ms, flip angle = 60°, acquisition voxel size = 1.8 × 1.7 × 8.0 mm^3^, and 30 phases per cardiac cycle.

**Fig. 1:**
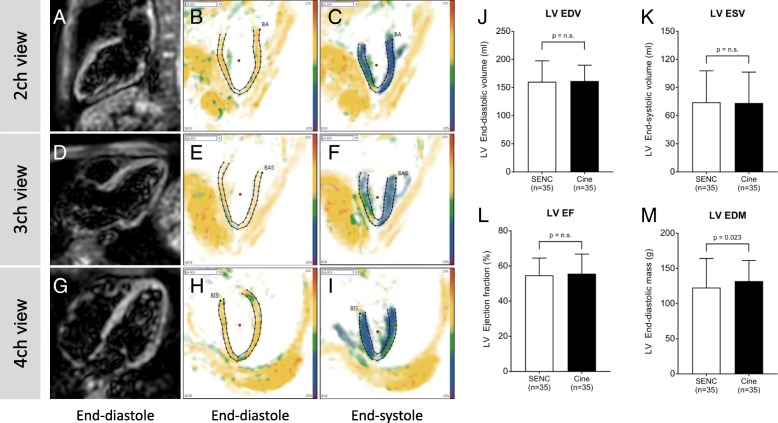
Example of fast-SENC CMR images derived in LV two-chamber (**a**), three-chamber (**d**) and four-chamber (**g**) views. All fast-SENC images were uploaded into a dedicated analysis software and endocardial and epicardial borders were traced at end-diastolic and end-systolic cardiac phases in LV two-chamber (**b** and **c**), three-chamber (**e** and **f**) and four-chamber (**h** and **i**) images to estimate LV volumes, EF and mass. Comparison of LVEDV (**j**), LVESV (**k**) and LVEF (**l**) between fast-SENC and conventional cine imaging did not show significant difference whereas LVEDM (**m**) was significantly underestimated by fast-SENC imaging. SENC = strain-encoded imaging; CMR = cardiac magnetic resonance; LV = left ventricle / ventricular; LVEDV = LV end-diastolic volume; LVESV = LV end-systolic volume; LVEF = LV ejection fraction; LVEDM = LV end-diastolic mass

A newly developed real-time free-breathing SENC imaging technique (Myocardial Solutions, Inc., Morrisville, North Carolina, USA) was employed to acquire fast-SENC images. Data were derived in three LV long-axis (two-, three- and four-chamber) views (Fig. 1a-i, respectively) and three short-axis planes at different LV levels (basal, mid-ventricular and apical). Relevant fast-SENC acquisition parameters were as follows: field-of-view = 256 × 256 mm^2^, slice thickness = 10 mm, voxel size = 4.0 × 4.0 × 10 mm^3^, single-shot spiral readout (3 interleaves) with acquisition time (TA) = 10 ms, flip angle = 30°, effective echo time (TE) = 0.7 ms, repetition time (TR) = 12 ms, temporal resolution = 36 ms, typical number of acquired phases = 22, spectrally selective fat suppression (SPIR), total acquisition time per slice < 1 s.

### Data analysis

Before starting the CMR data analysis, the observers were similarly trained by a representative of the software company with an emphasis on possible sources of error. All cine images were analyzed offline using Medis Suite, version 3.0 (Leiden, The Netherlands) or Virtue software (Morrisville, USA) in accordance to a recent consensus document for quantification of LV function and mass using CMR [1], while all fast-SENC images were uploaded from the scanner into a dedicated MyoStrain, version 4.2 software (Morrisville, USA). The end-diastolic and end-systolic cardiac phases were detected visually and after manual contouring of endocardial and epicardial borders LV end-diastolic (LVEDV), LV end-systolic (LVESV), LVEF and LV end-diastolic mass (LVEDM) were calculated. The LV longitudinal and circumferential strain was extracted from three LV short-axis and three LV long-axis fast-SENC images respectively. The global strain values were calculated by averaging measurements obtained from 16 segments for global longitudinal strain (GLS) and 18 segments for global circumferential strain (GCS).

### Statistics

Data analysis was performed using IBM SPSS Statistics version 21.0 software (SPSS Inc., Chicago, IL, USA) for Windows. Continuous variables were expressed as mean ± standard deviation. Differences in continuous variables were established using an unpaired Student t test or Wilcoxon signed rank test depending on their distribution. Pearson’s correlation coefficient was calculated to express the relation between the continuous variables. Bland-Altman analysis was performed to test if there was any bias in either CMR technique. Intraobserver and interobserver reproducibility for LVEF and LVEDM was quantified using intraclass correlation coefficient (ICC) and Bland-Altman analysis. Agreement was considered excellent for ICC > 0.74, good for ICC 0.60–0.74, fair for ICC 0.40–0.59, and poor for ICC < 0.40. Statistical significance was defined by a *p* value < 0.05.

## Results

### Demographic data

All study participants were able to complete the entire study protocol. Study participants with suspected or confirmed CAD were significantly older than the healthy volunteers (61.37 ± 10.93 y vs. 28.67 ± 4.89 y, *p* < 0.001). In the CAD group there were more male subjects (p < 0.001). Healthy volunteers had significantly lower body surface area (*p* = 0.026) and body mass index (p < 0.001). LVEDV and LVESV were similar in both groups, whereas LVSV and LVEF were significantly lower in CAD patients (LVSV: 80.48 ± 17.81 ml vs. 97.00 ± 18.96 ml, *p* = 0.021; LVEF: 52.09 ± 11.29% vs. 59.75 ± 1.36%, *p* = 0.034). Subjects with CAD had significantly higher LVEDM than healthy volunteers (143.96 ± 30.61 g vs. 82.00 ± 25.82 g, *p* < 0.001). Table 1 summarizes the demographic and LV functional parameters of the study population.

**Table 1:** Study participants’ characteristics

Parameter	Healthy volunteers(*n* = 12)	CAD patients(*n* = 23)	*P* value
Demographics			
Age (years)	28.67 ± 4.89	61.37 ± 10.93	< 0.001
Male gender	6 (50%)	21 (91%)	< 0.001
BSA (m^2^)	1.84 ± 0.22	1.99 ± 0.15	< 0.001
BMI (k/m^2^)	22.17 ± 2.45	26.84 ± 2.12	< 0.001
CAD	0 (0%)	23 (100%)	< 0.001
Volumetric and functional parameters			
LVEDV (ml)	162.33 ± 33.06	159.17 ± 39.98	0.694
LVESV (ml)	65.42 ± 14.52	78.78 ± 39.69	0.461
LVSV (ml)	97.00 ± 18.97	80.48 ± 17.81	0.021
LVEF (%)	59.75 ± 1.36	52.09 ± 11.29	0.034
LVEDM (g)	82.00 ± 25.82	143.96 ± 30.61	< 0.001
GLS (%)	-19.34 ± 1.28	−17.29 ± 3.17	0.034
GCS (%)	−20.21 ± 1.48	− 17.67 ± 2.63	0.001

Results are reported as mean ± standard deviation or total number (percentage). *CAD* coronary artery disease, *BSA* body surface area, *BMI* body mass index, *LV* left ventricle / ventricular; *LVEDV* LV end-diastolic volume, *LVESV* LV end-systolic volume, *LVSV* LV stroke volume, *LVEF* LV ejection fraction, *LVEDM* LV end-diastolic mass, *GLS* global longitudinal strain, *GCS* global circumferential strain

### Myocardial deformation analysis

As described in the methods, analysis of regional myocardial deformation was performed using three long-axis fast-SENC images (for GCS) and three short-axis fast-SENC images (for GLS). LV GLS and GCS were significantly lower in CAD patients than in healthy volunteers (GLS: − 17.29 ± 3.17% vs. − 19.34 ± 1.28, p = 0.034; GCS: -17.67 ± 2.63 ± − 20.21 ± 1.48%, *p* = 0.001) (Table 1).

### Intermethod agreement

**Fig. 2:**
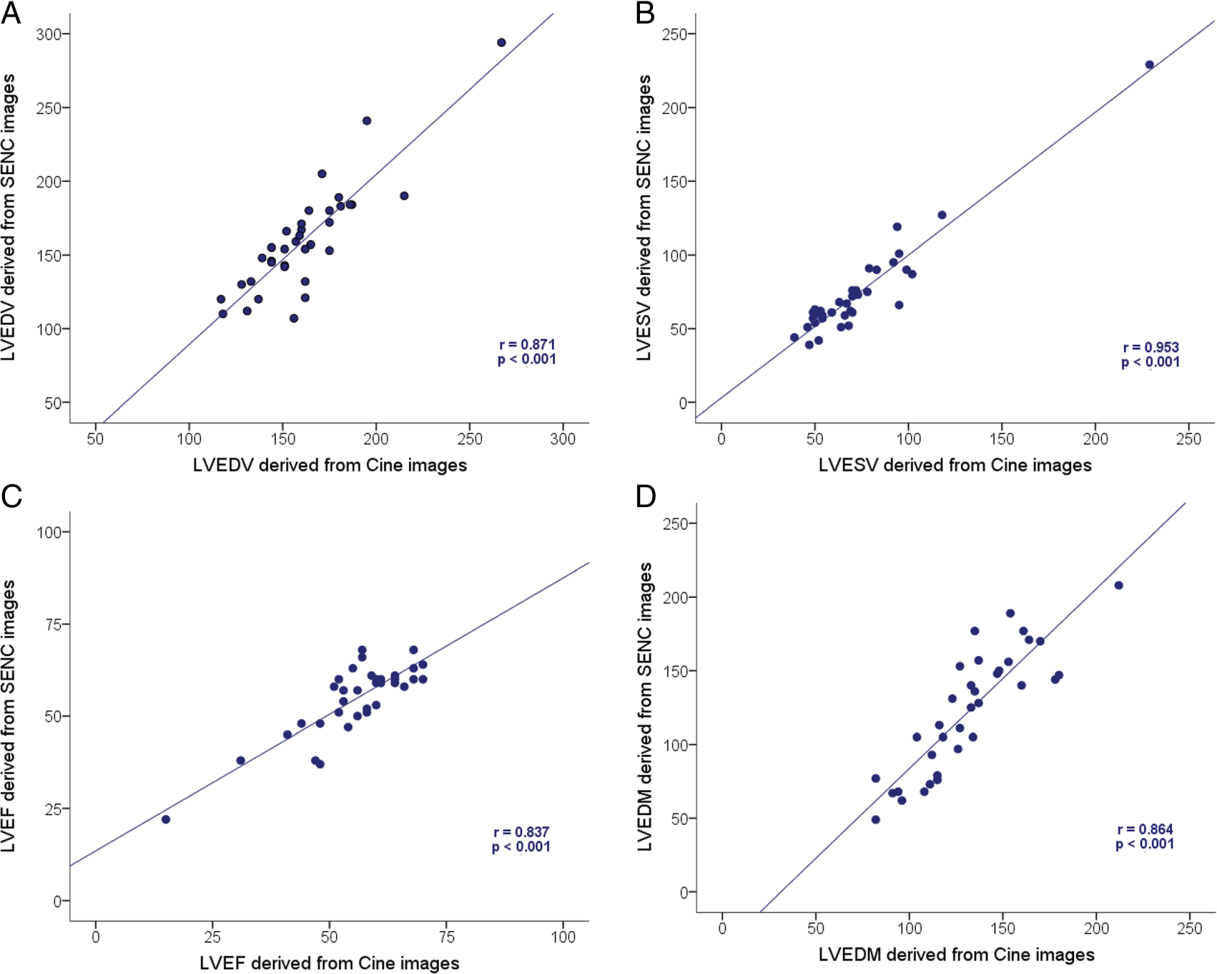
Correlation analysis of LVEDV (**a**), LVESV (**b**), LVEF (**c**) and LVEDM (**d**) between fast-SENC and conventional cine imaging. LV = left ventricle / ventricular; LVEDV = LV end-diastolic volume; LVESV = LV end-systolic volume; LVEF = LV ejection fraction; LVEDM = LV end-diastolic mass; SENC = strain-encoded imaging

**Fig. 3:**
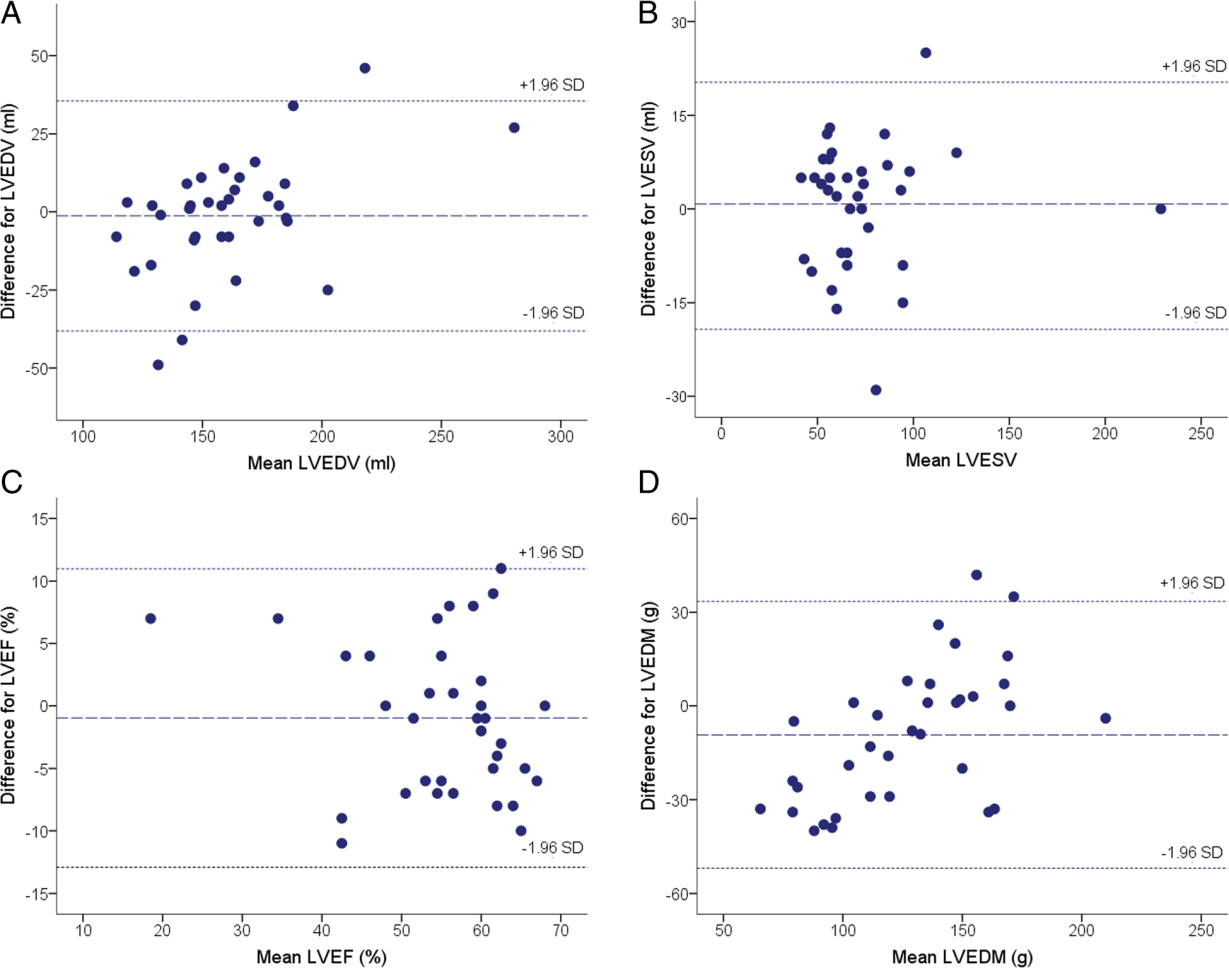
Bland-Altman plots with limits of agreement (1.96 SD) for LVEDV (**a**), LVESV (**b**), LVEF (**c**) and LVEDM (**d**). The middle-dashed line is the mean of difference of measures. The upper and lower dotted lines are 1.96 standard deviation. LVEDV = LV end-diastolic volume; LVESV = LV end-systolic volume; LVEF = LV ejection fraction; LVEDM = LV end-diastolic mass

SENC imaging and analysis were fast with a 15 s scan time and a 90 s post-processing time for a complete quantitative assessment. The LVEDV, LVESV and LVEF values derived from SENC images were similar as compared to those estimated using conventional cine imaging (160.26 ± 37.28 ml vs. 161.54 ± 28.17 ml, *p* = 0.928 for LVEDV; 74.20 ± 33.60 ml vs. 73.43 ± 33.04 ml, *p* = 0.620 for LVESV; and 54.71 ± 9.83% vs. 55.69 ± 11.11%, *p* = 0.374 for LVEF) (Fig. 1j, k and l). However, LVEDM measured in fast-SENC images was 7% lower when compared with estimated using cine images (122.71 ± 41.38 g vs. 131.94 ± 29.31 g, *p* = 0.023) (Fig. 1m). Accuracy analysis demonstrated significant correlation between fast-SENC and cine imaging techniques in the measurements of LVEDV (r = 0.871, *p* < 0.001), LVESV (r = 0.953, p < 0.001), LVEF (r = 0.837, p < 0.001) and LVEDM (r = 0.864, p < 0.001) (Fig. 2a, b, c and d respectively). The Bland-Altman analysis showed narrow limits of agreement (1.96 SD) for LVEDV (Fig. 3a), LVESV (Fig. 3b), LVEF (Fig. 3c) and LVEDM (Fig. 3d).

### Intraobserver and interobserver reproducibility

**Fig. 4:**
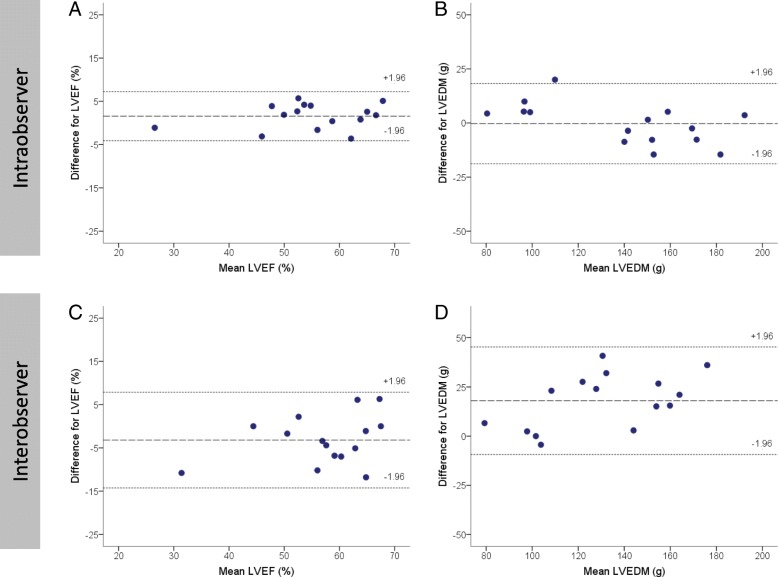
Bland-Altman analysis demonstrates the intraobserver (**a** and **b**) and interobserver (**c** and **d**) reproducibility of fast-SENC technique for LVEF (**a** and **c**) and LVEDM (**b** and **d**). The middle dashed line is the mean of difference of measures. The upper and lower dotted lines are 1.96 standard deviation. LVEF = LV ejection fraction; LVEDM = LV end-diastolic mass

There was excellent intraobserver agreement for LVEF: ICC 0.976 (0.918–0.992) and LVEDM: ICC 0.983 (0.949–0.994) derived using fast-SENC technique. The analysis of interobserver reproducibility also demonstrated excellent agreement, with slightly larger limits of agreement: ICC 0.895 (0.654–0.966) for LVEF and ICC 0.846 (0.157–0.958) for LVEDM. Figure 4 demonstrates Bland-Altman analysis of intraobserver and interobserver agreement for LVEF and LVEDM.

### Data acquisition and analysis time

The total scan time spent to acquire CMR images using fast-SENC was 15 s as only six heart beats without any breath-hold are necessary to derive 3 long-axis and 3 short-axis slices. In contrast, conventional cine imaging using bSSFP sequence in a patient with heart rate of 75 beats per minute lasted approximately 45–60 s per slice (30 phases per cardiac cycle including breathing commands and recovery period before the next breath-hold and following slice acquisition could be started). To acquire 3 long-axis and 3 short-axis images the same process was repeated 6 times (for every slice) with a total scan duration of 270–360 s.

The time spent for data post-processing using automated contour detection algorithm or machine learning software for fast-SENC (90 s) or bSSFP (120 s) was comparable. However, total time required for data acquisition and analysis was shorter for fast-SENC technique.

## Discussion

SENC is an advanced CMR technique for measuring regional myocardial function [4] as an alternative for time-consuming CMR tagging. The utility of SENC for the quantification of regional myocardial deformation has been previously validated in human [5, 6] and animal [7] studies. In previous studies the total scan duration for cine and SENC imaging was 30–40 s and for cine and CMR tagging imaging was 56–74 s. Importantly, the time spent for strain analysis per patient was significantly lower using SENC (4.1 min) when compared to CMR tagging (9.2 min, *p* < 0.001) [8]. A further step to reduce image acquisition time was the development of the fast-SENC, which is a real-time version of SENC that shortened the scan duration to a single heartbeat [7]. These achievements provided several advantages such as elimination of breath-holds or ability to capture dynamic processes such as onset of myocardial ischemia during the stress study or cardiac arrhythmias [7]. In our study we found that using fast-SENC technique image acquisition and post-processing can be performed in less than 2 min. There are no studies comparing the assessment of LV volumes, EF and mass using fast-SENC to other CMR imaging modalities. We did not find any relevant differences between fast-SENC and conventional cine CMR imaging regarding the estimation of LV volumes and LVEF, while fast-SENC significantly underestimated LV EDM. Recently we demonstrated that fast-SENC is a highly reproducible method for assessing LV strain [3]. In this study we found that intraobserver and interobserver agreement for LVEF and LVEDM measurements derived using fast-SENC technique is also excellent.

## Conclusion

The single heartbeat fast-SENC technique can be used as a good alternative to cine imaging for the precise calculation of LV volumes and ejection fraction in the interest of time-spent, especially in patients who are unable to perform breath-holds or with cardiac arrhythmias. However, LV mass is significantly underestimated with current fast-SENC technique. LVEF and LVEDM measurements derived using fast-SENC technique are highly reproducible.

## Abbreviations

CAD: Coronary artery disease
CMR: Cardiovascular magnetic resonance
EDM: End-diastolic mass
EDV: End-diastolic volume
EF: Ejection fraction
ESV: End-systolic volume
LV: Left ventricle / ventricular
SENC: Strain-encoded imaging
